# Fibroblast Growth Factor–21 ameliorates hepatic encephalopathy by activating the STAT3-SOCS3 pathway to inhibit activated hepatic stellate cells

**DOI:** 10.17179/excli2020-1287

**Published:** 2020-05-04

**Authors:** Yeboah Kwaku Opoku, Zhihang Liu, Justice Afrifa, Asare Kwame Kumi, Han Liu, George Ghartey-Kwansah, Harriet Koranteng, Xinghao Jiang, Guiping Ren, Deshan Li

**Affiliations:** 1 Department of Biology Education, Faculty of Science Education, University of Education, Winneba, Ghana; 2 Bio-pharmaceutical Laboratory, College of Life Sciences, Northeast Agricultural University, Harbin 150030, China; 3 Department of Medical Laboratory Science, University of Cape Coast, Cape Coast, Ghana; 4 Scientific Research Center, Second Affiliated Hospital of Harbin Medical University, Harbin, China; 5 Department of Protozoology, Institute of Tropical Medicine (NEKKEN), Nagasaki University, Japan; 6 Department of Biomedical Sciences, University of Cape Coast, Ghana; 7 Jiamusi University No. 148, Xuefu Road, Jiamusi, Heilongjiang, China

**Keywords:** acute liver failure (ALF), Thioacetamide (TAA), pro-inflammatory cytokines, fibrosis

## Abstract

Neurological dysfunction, one of the consequences of acute liver failure (ALF), and also referred to as hepatic encephalopathy (HE), contributes to mortality posing challenges for clinical management. FGF21 has been implicated in the inhibition of cognitive decline and fibrogenesis. However, the effects of FGF21 on the clinical and molecular presentations of HE has not been elucidated. HE was induced by fulminant hepatic failure using thioacetamide (TAA) in male C57BL/6J mice while controls were injected with saline. For two consecutive weeks, mice were treated intraperitoneally with FGF21 (3 mg/kg) while controls were treated with saline. Cognitive, neurological, and activity function scores were recorded. Serum, liver, and brain samples were taken for analysis of CCL5 and GABA by ELISA, and RT qPCR was used to measure the expressions of fibrotic and pro-inflammatory markers. We report significant improvement in both cognitive and neurological scores by FGF21 treatment after impairment by TAA. GABA and CCL5, key factors in the progression of HE were also significantly reduced in the treatment group. Furthermore, the expression of fibrotic markers such as TGFβ and Col1 were also significantly downregulated after FGF21 treatment. TNFα and IL-6 were significantly reduced in the liver while in the brain, TNFα and IL-1 were downregulated. However, both in the liver and the brain, IL-10 was significantly upregulated. FGF21 inhibits CXCR4/CCL5 activation and upregulates the production of IL-10 in the damaged liver stimulating the production pro-inflammatory cytokines and apoptosis of hepatic stellate cells through the STAT3-SOCS3 pathway terminating the underlying fibrosis in HE.

## Introduction

A healthy liver regulates its growth and homeostasis as to preserve constant tissue mass relative to metabolic stress in the body, thereby, prompting for a quick and vigorous cellular division of mature cells of the liver during damage (Galun and Axelrod, 2002[14]). This pathological condition induces hepatic inflammation and fibrosis as a natural response and healing mechanism to restore the damaged liver cells (Dooley and Ten Dijke, 2012[10]). However, the continuous formation, deposition, and transformation of parenchyma tissues to fibrotic tissues amidst inflammatory cytokines result in the loss of liver function or acute liver failure (Forbes and Rosenthal, 2014[12]). Myriads of factors can give rise to ALF, among which are viral infections, drug-induced liver injury, acute ischemic hepatocellular injury, neoplastic infiltration, acute Budd–Chiari syndrome, heatstroke, mushroom ingestion, and metabolic diseases such as Wilson’s disease (Schwartz et al., 2016[39]).

The clinical course of acute liver failure is that of progressive multi-organ failure with the severity of clinical signs and illness arising from the adverse metabolic consequences of loss of liver function, the systemic effect of toxins from the necrotic liver and the rate and degree of regeneration (Pyleris et al., 2010[32]). As part of the progressive multi-organ failure that can result from ALF, the function of the brain can also be affected, leading to hepatic encephalopathy (HE). HE occurs as a complication of advanced liver disease, either chronic or acute. Liver necrosis and sepsis cause the rise in blood cytokines which freely cross the blood-brain barrier resulting in brain edema (Jayakumar et al., 2015[18]). HE is defined as a range of neuropsychiatric abnormalities in patients with liver dysfunction and/or portosystemic shunting (PSS) after elimination of other recognized brain diseases, and is marked by intellectual impairments, personality changes, coupled with a depressed level of consciousness associated with multiple neurotransmitter systems, astrocyte dysfunction and cerebral perfusion to deep coma (Avraham et al., 2009[2]). Although the mechanisms causing these brain dysfunctions are still mostly unclear, a common pathogenic concept is that HE is caused by elements that under normal settings are efficiently processed by the liver, rather than insufficient production of substrates that could be essential for neurologic function (Cordoba, 2014[9]).

The prevalence and incidence of HE are linked to the severity of the underlying liver insufficiency (Riggio et al., 2011[35]). The prevalence of overt HE at the time of diagnosis of cirrhosis is 10 %-14 % in general with available data also indicate that overt HE will befall 30 %-40 % of patients with cirrhosis at a point through their clinical course and in most cases repeatedly in survivors (Jepsen et al., 2010[19]). Minimal HE (MHE) or covert HE (CHE) on the other hand occurs in 20 %-80 % of patients with cirrhosis (Bajaj, 2008[4]) while the prevalence of HE in pre-hepatic non-cirrhotic portal hypertension (PH) is however, not well defined (Vilstrup et al., 2014[43]).

Fibroblast growth factor-21 (FGF21), initially identified in mouse embryos, is a member of the subfamily 15/19 of the FGFs together with FGF19 and FGF23 in humans (Fon Tacer et al., 2010[11]). FGF21, a polypeptide of 209 amino acids, is mainly expressed in the liver (Inagaki, 2015[17]). However, under stress conditions, other tissue types, including white and brown adipose, muscle and pancreas also secrete it (Fon Tacer et al., 2010[11]). FGF21 has demonstrated great potential in the downregulation of TGF-β, NF-κB, phosphorylation of smad2/3 and IκBα in induced hepatic fibrotic mouse models (Xu et al., 2016[46]). Again, FGF21 increased the expression of Caspase-3, decreased the ratio of Bcl-2 to Bax, leading to the inactivation of hepatic stellate cells (HSCs) by inducing apoptosis. This, therefore, intimated an ameliorative effect on fibrogenesis. FGF21 has been reported to offer protection against liver damage caused by streptozotocin or hyperglycemia, provide neuroprotection and preserved cognitive function in mice through processes such as inhibition of oxidative stress, restoration of synaptic plasticity and mitochondrial brain function and increasing antioxidant activity (Ye et al., 2014[47]; Sa-nguanmoo et al., 2016[37]; Nehlin, 2017[28]). With these properties of FGF21 as the backdrop, coupled with the fact the management of HE is limited to the use of lactulose and antibiotics, which is however, not adequate. There is a need for research into novel therapeutic agents in the management of HE. Moreover, no study has been conducted to understand the effect of FGF21 on inflammatory cytokines, fibrotic markers, and clinical presentation of HE. This research, therefore, seeks to primarily elucidate the effect of FGF21 on encephalopathy with ALF as the precipitating factor.

## Materials and Methods

### Expression and purification of FGF21

The glycerol stock of *E. coli *Rosetta (DE3) expressing Recombinant pSUMO-hFGF21 plasmid was thawed and cultured with LB broth under ampicillin drug selection at 25^o^C for 16 hours in a shaking incubator until OD_600_ reached 0.4 to 0.6. Isopropyl-D-thiogalactoside (IPTG) was added to the culture medium to a final concentration of 0.25 mmol/L to induce FGF21 expression for 10 hours. The FGF21 protein was purified using Ni Sepharose 6 Fast Flow column (AKTA Purifier; GE Healthcare) and the purity of FGF21 analyzed by SDS-PAGE analysis.

### Animals experiment

Male C57BL/6J mice (6-8 weeks old) weighing 25 to 30 g were purchased from the Experimental Animal Center of Changchun Yisi Company. The mice were acclimatized and housed in standard-sized cages with enough nesting material under standard conditions of temperature and humidity (Temperature: about 23°C; relative humidity: about 50 %), pathogen-free room on a 12 h light cycle with access to food and water ad libitum. Using the rat model of acute liver failure (ALF) induced by thioacetamide (TAA) to mice (Zimmermann et al., 1989[50]), ALF was induced in 20 C57BL/6 mice by intraperitoneal injection with 200 mg·kg^-1^ of TAA (Sigma-Aldrich) and 10 C57BL/6 mice with normal sterile saline (NSS) as controls. In order to prevent hypovolemia, hypokalemia and hypoglycemia, 0.5 mL of a solution containing 5 % dextrose, 0.45 % NaCl, and 0.2 % KCl were injected subcutaneously in all mice 24 hours after TAA injection. The mice with induced ALF were subgrouped into two groups of 10 mice each. The first group of ALF mice together with the normal controls were treated with saline while the second group of ALF mice were treated with 3.0 mg/kg dose of FGF21 through i.p injection once daily for two weeks. All the animal experiments and protocols for this study were approved by the Animal Care and Use Committee of the Institute of Materia Medica, China.

### Assessment of neurological function 

Based on reflexes and task performance, neurological function was assessed on a 10-point scale (Chen et al., 1996[7]). These criteria included maintaining balance on a beam of width 1, 2 and 3 cm, ability to climb onto round and square poles, exit in less than 1 minute from a circle of 1 m in diameter, seeking, walking a straight line, startle reflex, grasping reflex, righting reflex, placing reflex and corneal reflex. For each task failed or for an abnormal reflex reaction observed, a score of 1 was assigned. Therefore, a higher score is an indication of poor neurological function. Neurological function was assessed one day after TAA injection and then divided into the various treatment groups with similar baseline neurological score after TAA injection. Post-treatment neurological score was also assessed at the end of the experiment.

### Assessment of activity 

Activity was assessed in an open field (20 X 30 cm field divided into 12 squares of equal size) (Fride and Mechoulam, 1993[13]). Two mice were concurrently observed for 5 min and locomotor activity recorded by counting the number of crossings at 1-minute intervals. Results were defined as the mean number of crossings per minute.

### Cognitive function 

Cognitive function studies were performed from day 8-12 after treatment with FGF21. A scaled-down form of the eight-arm maze earlier developed for rats was used to assess cognitive function (Pick and Yanai, 1983[31]). Water deprivation and a reward of 50 µL of water at the end of each arm was employed. Water consumption was limited overnight to achieve an appreciable level of water deprivation. The mice were placed into the maze and the number of entries until they entered all 8 arms or completed 24 entries counted. Once again mice were scored on a scale based on performance with lower scores indicative of better cognitive function. The data were calculated as the absolute number of correct choices made in the first eight to twelve choices of each test session. Using the area under the curve (AUC), the results are presented as previously reported (Pick and Yanai, 1983[31]).

### Histopathological studies

Mice were sacrificed by cervical dislocation and serum, liver, and brain samples collected. The liver and brain samples were flash-frozen in liquid nitrogen and stored at −80 °C for further analysis. Some portion of the liver was preserved in buffered formaldehyde and embedded in paraffin. Tissue sections (5 μm) were cut and stained with hematoxylin and eosin for histological observation.

### Measurement of CCL5 and GABA

Snap-frozen liver and brain samples were incubated for 30 minutes in 1 mL phenylmethylsulfonyl fluoride (PMSF) with proteinase inhibitor for isolation of total protein. C-C Motif Chemokine Ligand 5 (CCL5) chemokine levels in the serum, liver, brain and Gamma-Aminobutyric Acid (GABA) neurotransmitter in serum and brain samples were measured and quantified using commercially available ELISA kits (R&D Systems, USA) in accordance with the manufacturer’s protocol and guidelines. Samples were measured at 450 nm on ELISA plate reader (BioTek, USA) followed with cytokines quantification.

### RNA isolation and Real-time PCR

The liver and brain samples were homogenized using Tissuelyser-24 (Jingxin Co., Ltd., Shanghai, China), and total RNA was isolated with Trizol (Invitrogen). cDNA synthesis was performed using M-MLV reverse transcriptase (Promega). The cDNA was then analyzed by Real-time quantitative PCR using iTaq SYBR Green Supermix (Bio-Rad, Hercules). The primers for the Real-time quantitative PCR are listed in Table 1(Tab. 1).

### Statistical analysis

Data was analyzed with GraphPad Prism for windows. Mean ± SD or SEM values were calculated, and one-way ANOVA was used to test for significance at p<0.05 followed with Tukey’s Post hoc analysis for comparison between groups.

## Results

### FGF21 restores neurological and cognitive functions in mice with HE

**Figure 1:**
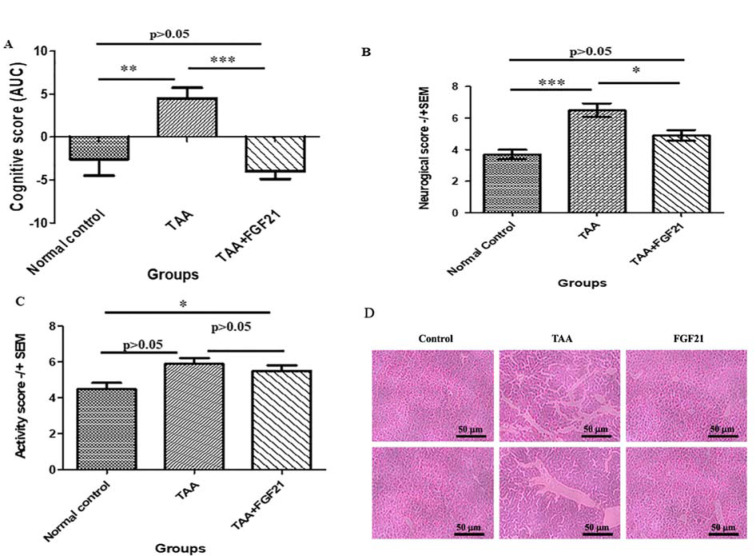
FGF21 protects mice with acute hepatic encephalopathy from cognitive dysfunction and liver fibrosis. Hepatic encephalopathy was induced by ALF in 20 C57BL/6 mice by intraperitoneal injection of 200 mg·kg^-1^ of TAA and 10 C57BL/6 mice with normal sterile saline (NSS) as controls. The HE-induced mice were subgrouped into two groups of 10 mice each. The first group, together with the normal controls, were treated with saline while the second group was treated with 3.0 mg/kg dose of FGF21 through i.p injection once daily for two weeks. The effect of FGF21 on cognitive, neurological, and activity functions was assessed. A. Cognitive score was assessed by the 8 armed-maze from day 8-12 and the results presented as the area under the curve. B. Neurological score was assessed based on reflexes and task performance two days after induction of hepatic failure and 12 days after treatment and data are presented as mean ± SEM. C. Activity score was assessed in an open field (20 x 30 cm) divided into 12 squares of equal size, and data are presented as mean ± SEM. D. Haematoxylin and eosin staining of the liver. All statistical analysis was performed using ANOVA, followed by Tukey’s multiple comparison tests. The level of significance is indicated by p>0.05 (not significant), *P<0.05, **P<0.01, ***P>0.001.

There was a significant increase in the cognitive score after administration of TAA compared to the normal control group (one-way ANOVA, F=8.206, p=0.0016). However, a significant restoration in the cognitive score was observed after treatment with FGF21. With Tukey’s multiple comparison tests, significant differences between Normal control vs. TAA (q=5.347, p<0.05) and TAA vs. TAA+FGF21 (q=4.456, p<0.05) were revealed. For the neurological score, a similar trend was observed with the administration of FGF21 again reducing the high neurological score induced by TAA (one-way ANOVA, F= 15.01, p<0.0001). Comparison with Tukey’s again showed significant differences between Normal control vs. TAA (q=7.722, p<0.05) and TAA vs. TAA+FGF21 (q=4.413, p<0.05). Although a significant difference was detected in the activity score (one-way ANOVA, F=5.032, p<0.0001), a comparison of the groups revealed no significant differences between normal control vs. TAA+FGF21 (q=3.111, p>0.05) and TAA vs. TAA+FGF21 (q=1.244, p>0.05) (Figure 1(Fig. 1)).

### FGF21 treatment abates CCL5 (RANTES) chemokine and gamma-aminobutyric acid (GABA) in HE-induced mice

**Figure 2:**
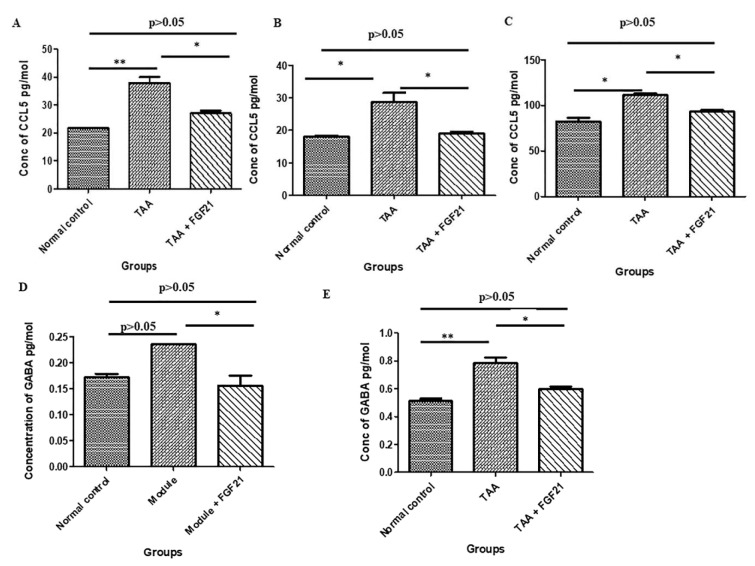
FGF21 regulates CCL5 chemokine and GABA neurotransmitter in the brain, liver, and serum of mice with acute hepatic encephalopathy. HE-induced mice were treated i.p with FGF21 (3mg/kg) while HE and normal controls were treated with saline for two weeks. Serum, liver, and brain samples were collected on the day of sacrifice. CCL5 chemokine levels in the serum, liver, brain and GABA neurotransmitter in serum and brain samples were measured and quantified using commercially available ELISA kits (R&D Systems, USA). A. CCL5 level in the brain. B. CCL5 level in the liver CCL5. C. CCL5 level in the serum. D. GABA levels in the brain. E. GABA levels in the serum. All statistical analysis was performed using ANOVA, followed by Tukey’s multiple comparison tests. The level of significance is indicated by p>0.05 (not significant), *P<0.05, **P<0.01, ***P>0.001.

There was a significant increase in CCL5 concentration in the brain (p<0.001), liver (p< 0.0322) and serum (p<0.05) of HE-induced mice compared to the healthy controls with no significant difference between TAA+FGF21 and healthy controls (p>0.05). Nevertheless, a significant reduction in the levels of CCL5 was observed after FGF21 treatment. Tukey’s multiple comparison tests revealed significant differences between normal control vs. TAA (p<0.05) and TAA vs. TAA+FGF21 (p<0.05) in the brain liver and serum. The concentration of GABA in the brain was also significantly elevated after treatment with TAA. Administration of FGF21, however, was marked by a reduction comparable to the levels in the normal controls. With the exception of TAA vs. TAA+FGF21, which was significant, all other comparisons by Tukey’s post hoc analysis were insignificant (p>0.05). GABA concentration in the serum was also significantly increased after administration of TAA with significant differences both between normal control vs. TAA (p<0.01) and TAA vs. TAA+FGF21 (p<0.05). However, no significant difference was recorded between normal controls, and FGF21 treated group (p>0.05) (Figure 2(Fig. 2)).

### FGF21 down-regulates fibrotic markers in hepatocyte of the HE induced mice

**Figure 3:**
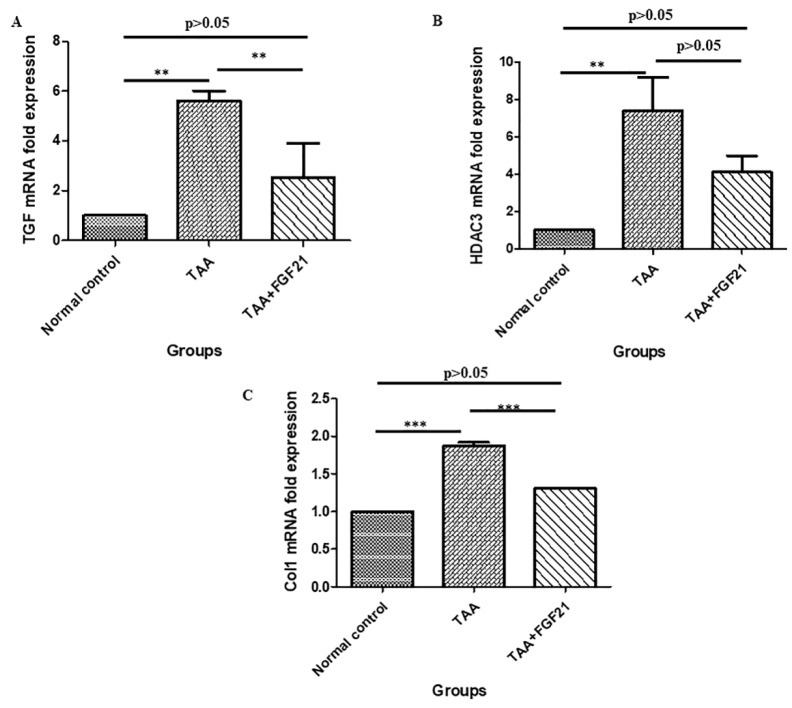
FGF21 downregulates fibrotic markers in hepatocyte of the mice with hepatic encephalopathy. HE-induced mice were treated i.p with FGF21 (3 mg/kg) while HE and normal controls were treated with saline for two weeks. Liver samples were harvested on the day of sacrifice, homogenized, and total RNA isolated with Trizol. Using real-time quantitative PCR, the mRNA expressions of fibrotic markers were analyzed relative to the expression β-actin and calculated as 2^−ΔΔCt^. A. TGF-β1. B. HDAC3. C. Col1. All statistical analysis was performed using ANOVA, followed by Tukey’s multiple comparison tests. The level of significance is indicated by p>0.05 (not significant), *P<0.05, **P<0.01, ***P>0.001.

The expression of TGF-β1 in the FGF21 treated mice was significantly reduced compared to TAA mice without treatment (p<0.001). Also, a significant upregulation of TGF-β1 in the TAA mice without treatment compared to the normal controls was recorded (p<0.0001) while TGF-β1 expression was reduced to statistically similar levels between the FGF21 treated mice and the normal controls (p>0.05). Similarly, the expression of Col1 was significantly reduced in the FGF21 treated mice compared to the TAA group without treatment (p<0.0001) whereas there was upregulation of Col1 in the TAA mice without treatment compared to the normal controls (p<0.0001). Again, there was a decrease in HDAC3 in the FGF21 treated mice compared to the TAA mice without treatment. However, it did not reach statistical significance (p>0.05) (Figure 3(Fig. 3)).

### FGF21 downregulates the expression of proinflammatory cytokines in the brain and hepatocyte of HE-induced mice

**Figure 4:**
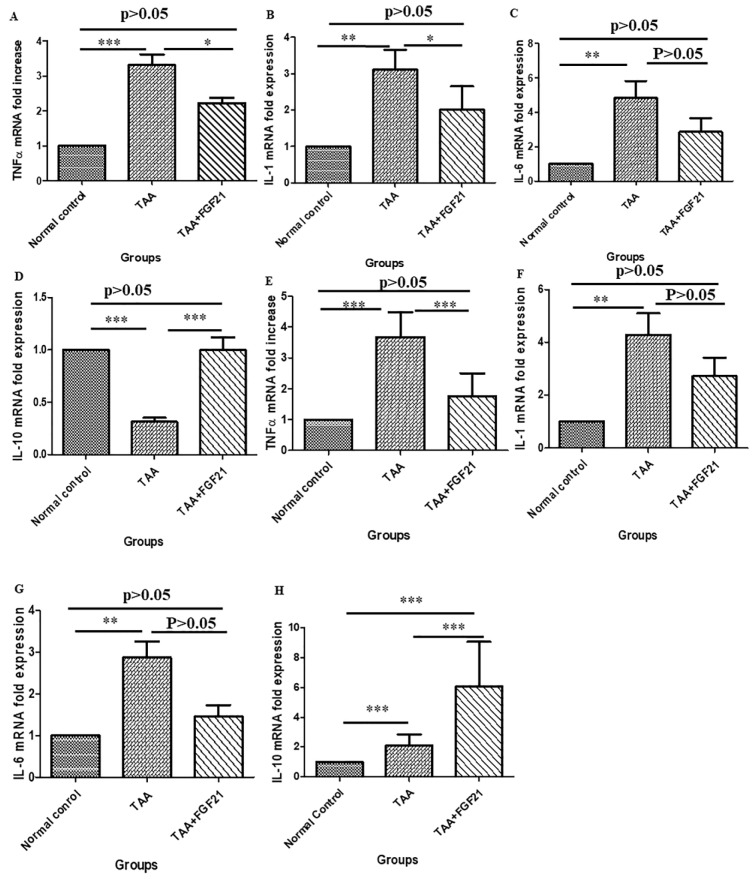
FGF21 suppresses the expression of pro-inflammatory cytokines in the brain and the liver of HE-induced mice. HE-induced mice were treated i.p with FGF21 (3 mg/kg) while HE and normal controls were treated with saline for two weeks. Liver and brain samples were collected on the day of sacrifice, homogenized, and total RNA isolated with Trizol. The mRNA expressions of pro-inflammatory markers were analyzed by Real-time quantitative PCR relative to the expression of β-actin and calculated as 2^−ΔΔCt^. In the brain; A. TNF-α. B. IL-1β. C. IL-6 D. IL-10. In the liver; E. TNF-α. F. IL-1β. G. IL-6. H. IL-10. All statistical analysis was performed using ANOVA, followed by Tukey’s multiple comparison tests. The level of significance is indicated by p>0.05 (not significant), *P<0.05, **P<0.01, ***P>0.001.

In the brain, the expression of TNF-α and IL-1 were significantly downregulated in the FGF21 treated mice. There were significant differences between FGF21 treated mice and TAA-induced mice without treatment (p<0.05) for both TNF-α and IL-1 while significant differences of p<0.001 and p<0.01 were recorded between normal controls vs. TAA group respectively for TNF-α and IL-1. On the other hand in the liver, a significant reduction in the expression of TNF-α and IL-6 in the FGF21 mice compared to the TAA mice without treatment (p<0.001) was observed, whereas TNF-α (p<0.001), IL-6 (p<0.001) and IL-1 (p<0.0001) were upregulated in the TAA group compared to the normal controls. However, there was no significant difference among the normal controls and the FGF21 treated mice (P>0.05) in both the brain and liver for TNF-α, IL-1, and IL-6. Again, both in the brain and the liver, there was significant upregulation of the expression of IL-10 in the FGF21 treated mice compared to both the TAA mice without treatment and the normal controls (Figure 4(Fig. 4)).

## Discussion

There is a great impact on the quality of life of patients with chronic liver disease in spite of the apparent lack of clinical signs or symptoms in minimal HE (Schomerus and Hamster, 2001[38]). Common strategic measures for chronic HE such as withdrawal of dietary protein, lactulose and neomycin have, however, proved futile in the treatment and management of severe acute HE (Pyleris et al., 2010[32]). Hence there is the need for novel therapeutics for the management of HE. Overt HE involves a plethora of episodic mental and motor disorders lasting from hours to days in a previously mentally stable patient (Bajaj et al., 2011[3]). The administration of TAA induces acute liver failure leading to changes in the central nervous system (CNS) comparable to those seen in HE (Zimmermann et al., 1989[50]; Avraham et al., 2009[2]). The hepatotoxicity of TAA has been attributed to the generation of free radicals and oxidative stress. The influx of cytokines into the CNS during HE causes brain edema or neuroinflammation in mice (Bémeur and Butterworth, 2013[5]). Furthermore, the infiltration of inflammatory cells into the CNS results in the damage of nerves in the brain tissues leading to neurological dysfunction and impaired cognitive function in HE mice (Glass et al., 2010[16]). In this experiment, administration of TAA led to an increase in cognitive score. However, upon treatment with FGF21, the scores were significantly lower compared to the normal controls. This is, however, not surprising as the effect of FGF21 on cognitive function has been demonstrated in a number of studies (Sa-Nguanmoo et al., 2016[37]). Hence this report emphasizes the fact that this cognitive effect of FGF21 can be capitalized on in the management of HE. Similar trend was again recorded for the neurological score with a significant difference between the FGF21 treated group and the model group. FGF21 treatment, however, did not reduce the activity score as there was no significant difference after treatment.

Liver damage strongly activates stellate cells to secrete chemokines CCL5 which recruits T cells, eosinophils, dendritic cells, mast cells, NK cells, and basophils to the sites of inflammation by interacting with CCR1, CCR3, and CCR5 receptors (Marques et al., 2013[24]; Choi et al., 2016[8]). The binding of CCL5 to CCR1 and CCR5 receptors in the liver has been demonstrated to promote hepatic fibrosis in mice (Moreno et al., 2005[27]). The attenuation of CCL5, however, causes the amelioration of liver fibrosis (Nellen et al., 2012[29]). In this study, haematoxylin and eosin staining showed restoration of the hepatic cell integrity of the mice treated with FGF21 comparable to the healthy control mice, indicative of the inactivation of hepatic stellate cells (Figure 1(Fig. 1)). To understand the mechanism underlying this restoration, we quantified the amount of CCL5, and it was observed that FGF21 significantly reduced the levels of CCL5 in the serum, liver, and the brain. Ammonia is one of the key factors in the pathogenesis of HE. At appreciable levels of 0.15-0.75 mM, it has been demonstrated to escalate GABA-induced chloride current in cultured neurons via the modification of the affinity of the GABA(A) receptor for GABA (Jones, 2002[20]). According to Albrecht and Jones (1999[1]), HE with chronic liver disease as the precipitating factor usually presents an imbalance between excitatory and inhibitory neurotransmitters. FGF21 administration was associated with a significant reduction in GABA in the serum and brain. FGF21, therefore, could be acting as GABA(A) receptors agonist and preventing pseudo-bile ductules and canaliculus formation in the liver, similar to that of GABA(A) receptor agonist muscimol in rats injected with GalN (Wang et al., 2016[45]) as shown in the H&E liver staining (Figure 1D(Fig. 1)).

The induction of HSCs during hepatic injury results in the secretion and accumulation of excessive extracellular matrix (ECM) deposition into the hepatic interstitium, a cause of liver fibrosis (Giannandrea and Parks, 2014[15]). The ECM in liver fibrosis is maintained by transforming growth factor β1 (TGF-β1) by enhancing the activation of fibroblasts and HSCs while inhibiting apoptosis of fibronectin, collagen (Col) types I and III, tenascin, elastin and osteonectin, the factors involved in liver fibrogenesis (Karin et al., 2016[21]). The dysregulated biosynthetic of accumulated ECM in the liver cells leads to the formation of pseudo-bile ductules and enlarged canaliculus (Zhang et al., 2016[49]). Moreover, the over-expression of the active form of TGF-β1 in the liver of transgenic mice is enough to induce fibrotic disease in multiple organs, even including the kidney (Sanderson et al., 1995[36]). FGF21 downregulated the expression of both TGF-β1 and Col1, resulting in the amelioration of the encephalopathy. The significant expression of both markers can be attributed to the fact that TGF-β1-induces the expression of type I collagen (Meng et al., 2016[25]). Studies have shown that regulation of factors precipitating HE is vital in the management of HE as about 90 % of patients are restored upon correction of these factors (Strauss et al., 1992[41]). Therefore, the attenuation of fibrosis, the precipitating factor of HE by FGF21 better explains the ameliorative effect observed. Although recent studies have implicated HDACs in multiple organ fibrosis (Pang and Zhuang, 2010[30]), HDAC3 was not significantly downregulated in this research. This goes a long way to endorse the anti-fibrotic potential of FGF21 previously reported (Xu et al., 2016[46]).

Cognitive dysfunctions and neurological disturbance that arise from the development of brain edema in HE in mice have been associated with increased ammonia and proinflammatory cytokines in the liver and the brain (Jayakumar et al., 2015[18]). Increase in ammonia concentration is toxic to astrocytes and generates reactive oxygen or nitrogen oxide species stimulating an inflammatory response in HE (Steele and Robinson, 2012[40]). Considering the role of proinflammatory cytokines in liver fibrosis, cognitive dysfunction, and neurological disturbance in HE, we quantified their expression in the liver and brain. FGF21 significantly downregulated the expression of proinflammatory cytokines both in the brain and the liver. These observations were in agreement with the observed improvement in cognitive, neurological activities, and the down-regulation of fibrotic markers. This observation, hereby, suggests that FGF21 treatment blocks portosystemic‐shunting (PSS) by downregulating proinflammatory cytokines. 

**Figure 5:**
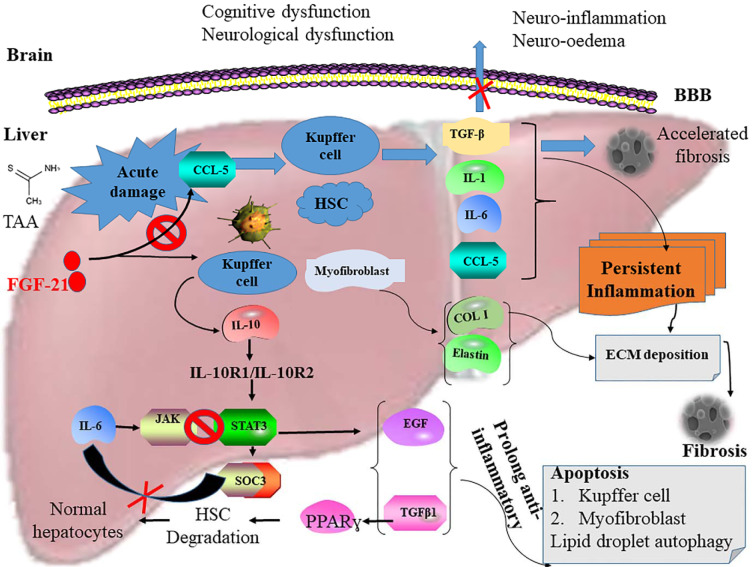
Diagrammatic presentation of the activation of the STAT3-SOCS3 signaling pathway and inhibition of CXCR4/CCL5 interaction by FGF21 in HE-induced mice. The activation of CXCR4/CCL5 in liver damage stimulates Kupffer cells and activated HSCs to secrete TGFβ, IL-1, IL-6, and RANTES, subsequently leading to the stimulation and acceleration of persistent inflammation and fibrosis. This enhances excessive extracellular matrix (ECM) deposition in the hepatocytes. Also, myofibroblast secretes excess Col1 and elastin into the ECM, resulting in the formation of fibrosis. Proinflammatory cytokines and chemokines also cross the blood-brain barrier to cause neuroinflammation in the brain resulting in cognitive and neurological dysfunction in acute hepatic encephalopathy (AHE). However, FGF21 blocks the activation of CXCR4/CCL5 and associated pathway resulting in the rapid resolution of fibrosis and inflammation culminating in the restoration of cognitive and neurological dysfunction in HE. Synergistically, FGF21 also upregulates the expression of IL-10 thereby activating STAT3. This leads to the stimulation of SOCS3 to prolong the expression of anti-inflammatory response through the secretion of TGFβ1 and EGF. These facilitate the induction of apoptosis in the Kupffer cells and myofibroblast and increase autophagy of the lipid droplets in the damaged hepatocytes. SOCS3 inhibits IL-6 to block the JAK-STAT3 signaling pathway. TGFβ1 also stimulates PPARγ-mediated degradation of activated HSCs to restore normal hepatocytes in HE-induced mice as seen in the H&E staining of hepatocytes of the HE mice treated with FGF21.

The expression of IL-10 was upregulated in the liver after FGF21 treatment suggestive of the induction of antiapoptotic and antioxidant molecules to inhibit oxidative stress in the diseased organ. The underlying liver fibrosis in HE increases the permeability of the blood-brain barrier allowing the infiltration of proinflammatory cytokines into the brain (Figure 5(Fig. 5)) (Wang et al., 2010[45]). However, FGF21 treatment again increased the expression of anti-inflammatory IL-10 in the brain. This, therefore, suggests an indirect protective role by FGF21in the amelioration of HE through the stimulation of signal transducer and activator of transcription 3 (STAT3) signaling pathway similar to that of recombinant IL-22 treatment in liver injury and alcoholic fatty liver (Kong et al., 2013[22]).

The high expression of Col1, TGFβ, IL-6 in the HE induced mice is an indication of an increase in turnover of ECM due to myofibroblasts and HSCs in the damaged liver (Marin et al., 2017[23]). IL-6 is a regulator of STAT3 phosphorylation mediated by receptor-associated Janus tyrosine kinases (JAKs) (Berishaj et al., 2007[6]). The IL-6-JAKs-STAT3 pathway promotes proinflammation, HSCs activation, regulates fibroblast cell growth, survival, and regeneration leading to liver fibrosis (Yu et al., 2009[48]). However, FGF21 treatment was marked with a significant decrease in fibrotic markers and chemokines, such as TGFβ, Col1, IL-6, IL-1, thereby preventing the formation of pseudo-bile ductules and enlarge canaliculus in the liver. These findings indicate that FGF21 treatment severely inhibits the JAK pathway in HE-induced mice. The observed significant upregulation of IL-10 in FGF21 treated mice also serves as an indicator of the activation of STAT3 by IL-10 and subsequent upregulation of the expression of suppressor of cytokine signaling 3 (SOCS3) in macrophages (Qin et al., 2012[34]). The activation of SOCS3 inhibits JAKs pathway by binding gp130 of IL-6 (Tamiya et al., 2011[42]). The STAT3-SOCS3 pathway induces a prolonged anti-inflammatory response (Qin et al., 2016[33]). The SOCS3 pathway induces apoptosis in Kupffer and myofibroblast cells and stimulates autophagy of lipid droplets in the injured liver (Mezale et al., 2017[26]). FGF21 synergistically blocks CXCR4/CCL5 interaction on the hepatocytes to inhibit the production of TGFβ, IL-6, IL-1, RANTES, Col1, and elastin. This blockage prevents chemokines and proinflammatory cytokines from crossing the BBB, inactivating excessive ECM deposition and terminating liver fibrosis. At the same time, FGF21 upregulates the expression of IL-10 to activate the STAT3-SOCS3 pathway leading to the restoration of normal liver homeostasis (Figure 5(Fig. 5)).

In conclusion, FGF21 induces IL-10 to activate the STAT3-SOCS3 pathway, thereby inhibiting IL-6-JAKs-STAT3 pathway leading to the restoration of normal liver homeostasis in acute HE mice. Again, FGF-21 blocks CXCR4/CCL5 activation in the damaged liver cells inhibiting Kupffer cells, activated HSCs, and fibrotic cells from producing TGFβ, IL-6, IL-1, RANTES, Col1, and elastin. This terminates fibrosis, infiltration of inflammatory cells in the liver and blocks chemokines and interleukins from crossing the BBB membrane, thereby restoring cognitive and neurological function in HE-induced mice. FGF21 has a remarkable liver regeneration effect in acute hepatic encephalopathy in mice. Therefore, its therapeutic properties can be exploited in the management of HE with further research warranted.

## Conflict of interest

Authors have declared that no competing interests exist.

## Figures and Tables

**Table 1:**
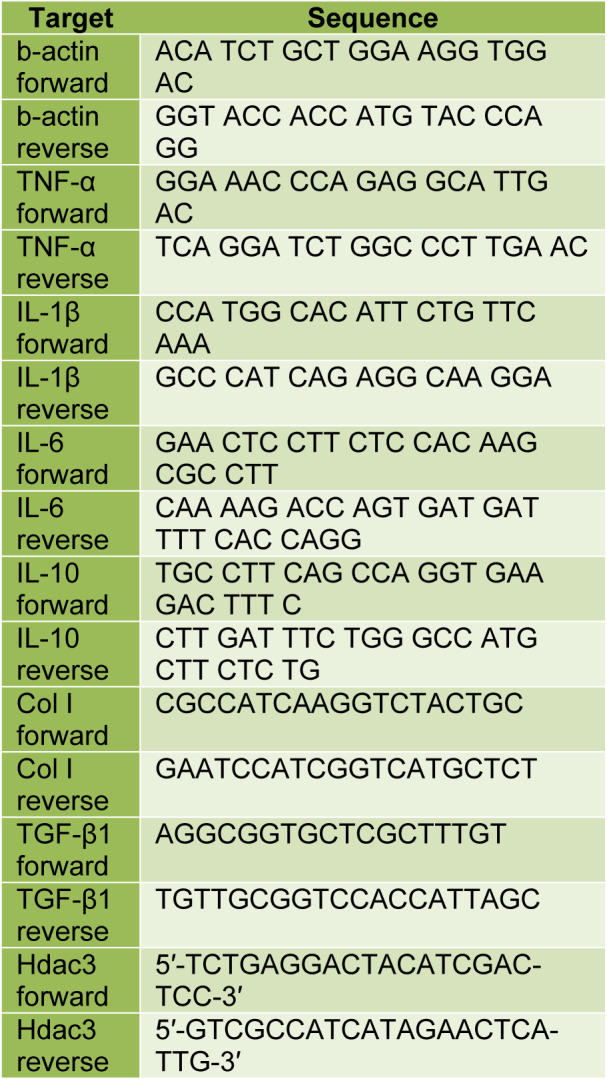
Primer sequences for real time quantitative PCR
